# miRNA‐1 regulation is necessary for mechanical overload‐induced muscle hypertrophy in male mice

**DOI:** 10.14814/phy2.70166

**Published:** 2025-01-06

**Authors:** Shengyi Fei, Blake D. Rule, Joshua S. Godwin, C. Brooks Mobley, Michael D. Roberts, Ferdinand von Walden, Ivan J. Vechetti

**Affiliations:** ^1^ Department of Nutrition and Health Sciences University of Nebraska‐Lincoln Lincoln Nebraska USA; ^2^ School of Kinesiology Auburn University Auburn Alabama USA; ^3^ Department of Women's and Children's Health Karolinska Institutet Stockholm Sweden

**Keywords:** hypertrophy, miR‐1, skeletal muscle

## Abstract

MicroRNAs (miRNAs) are small, noncoding RNAs that play a critical role in regulating gene expression post‐transcriptionally. They are involved in various developmental and physiological processes, and their dysregulation is linked to various diseases. Skeletal muscle‐specific miRNAs, including miR‐1, play a crucial role in the development and maintenance of skeletal muscle. It has been demonstrated that the expression of miR‐1 decreases by approximately 50% in response to hypertrophic stimuli, suggesting its potential involvement in muscle hypertrophy. In our study, we hypothesize that reduction of miR‐1 levels is necessary for skeletal muscle growth due to its interaction to essential pro‐growth genes. Promoting a smaller reduction of miR‐1 levels, we observed a blunted hypertrophic response in mice undergoing a murine model of muscle hypertrophy. In addition, our results suggest that miR‐1 inhibits the expression of *Itm2a*, a membrane‐related protein, as potential miR‐1‐related candidate for skeletal muscle hypertrophy. While the exact mechanism in muscle hypertrophy has not been identified, our results suggest that miR‐1‐regulated membrane proteins are important for skeletal muscle hypertrophy.

## Graphical abstract

Schematic representation of mice subjected to mechanical overload (via synergist ablation) following miR‐1 overexpression to determine its role in skeletal muscle hypertrophy. Muscle tissue samples were collected and analyzed through a combined approach integrating RNA‐seq to identify global gene expression changes and enhanced crosslinking immunoprecipitation sequencing (eCLIP‐seq) to map miRNA‐mRNA interactions. Our results suggest that miR‐1 regulation is necessary for muscle hypertrophy through mechanotransduction pathways.

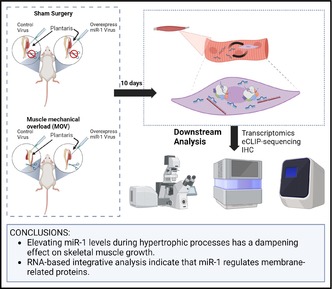

## 1 INTRODUCTION

Skeletal muscle hypertrophy is a complex process involving well‐characterized mechanisms, such as enhanced mammalian target of rapamycin complex 1 (mTORC1) signaling, ribosome biogenesis, elevations in muscle protein synthesis rates, and more recent mechanisms, such as extracellular matrix remodeling, mechanotransduction‐based signaling, epigenetic changes, and microRNA regulation (Roberts et al., 2023).

MicroRNAs (miRNAs) are small noncoding RNAs that are crucial for regulating gene expression through post‐transcriptional mechanisms. They achieve this regulation by base pairing with the 3′ untranslated region (3′ UTR) of target messenger RNA (mRNA) molecules, and their dysregulation has been linked to various diseases (Marceca et al., 2020; O'Brien et al., 2018). Muscle‐specific/enriched miRNAs (myomiRs) play significant roles in the development and functional adaptation of skeletal and cardiac muscles (McCarthy, 2008; Sempere et al., 2004). For example, McCarthy and Esser were the first to report changes in the expression of myomiRs (i.e., miR‐1 and miR‐133a) in adult skeletal muscle hypertrophy (McCarthy & Esser, 2007). Specifically, they observed a decrease in myomiR levels of approximately 50% in response to mechanical overload (MOV)‐induced muscle hypertrophy, which was later confirmed by qPCR by the same group (Chaillou et al., 2013). These results and the evidence showing that miR‐1 targets pro‐growth genes such as IGF‐1 and cyclin D (Elia et al., 2009; Leone et al., 2011; Li et al., 2012) suggest that miR‐1 acts as a “molecular break” on muscle hypertrophy. Indeed, reduction of miR‐1 following load‐induced muscle hypertrophy has been corroborated in rodents (Vechetti et al., 2019, 2021) and humans (Drummond et al., 2008; D'Souza et al., 2019) studies; however in humans, miR‐1 downregulation occurs in acute bouts of exercise with aminoacids/protein ingestion but not with long periods of resistance training nor in aged skeletal muscle (Davidsen et al., 2011; Drummond et al., 2008; D'Souza et al., 2019).

Although these results strongly suggest an essential role for miR‐1 in skeletal muscle hypertrophy, the mechanisms associated with this process are not completely understood. To test the hypothesis that miR‐1 downregulation is necessary for skeletal muscle hypertrophy, we overexpressed miR‐1 (lentivirus injection) in mice that underwent 10 days of MOV‐induced muscle hypertrophy. Our findings underscore the importance of miR‐1 regulation during muscle hypertrophy, showing that preventing the reduction in miR‐1 attenuates the hypertrophic responses promoted by MOV. In addition, by integrating RNA‐seq and ECLIP‐seq analyses, we identified integral membrane protein 2A (*Itm2a*), a membrane‐related protein, as potential player in the hypertrophic response induced by MOV. These findings provide new insights into the mechanisms underlying muscle hypertrophy and may have therapeutic implications in muscle‐related disorders.

## 2 MATERIALS AND METHODS

### 2.1 Animals

All animal procedures were conducted in accordance with the institutional guidelines for the care and use of laboratory animals, as approved by the Institutional Animal Care and Use Committee of the University of Nebraska‐Lincoln. Adult (5 months of age) male C57BL/6J (obtained from The Jackson Laboratory) mice were housed in a temperature‐and humidity‐controlled room and maintained on a 12‐h light–dark cycle with food (Teklad Global 16% Protein Rodent Diet, Inotiv) and water ad libitum. Animals were sacrificed via CO_2_ asphyxiation, followed by cervical dislocation.

### 2.2 Mechanical overload

Mice underwent 10 days of either a sham or a bilateral synergist ablation surgical procedure (mechanical overload, MOV), as previously described (Chaillou et al., 2013; Figueiredo & McCarthy, 2019; Vechetti Jr et al., 2021). Briefly, mice were anesthetized using isoflurane under sterile conditions, and a small incision was made on the dorsal aspect of the hindlimb, and about half of the gastrocnemius‐soleus complex was carefully removed without disturbing the blood supply or innervation to the plantaris muscle; the sham surgery involved all aspect of the synergist ablation surgery without excising the muscles. A total of 10 mice were used, with five mice undergoing bilateral sham and five MOV surgeries. The plantaris muscles were excised 10 days after the surgical procedures, weighed, flash‐frozen in liquid nitrogen, and stored at −80°C until downstream analysis. The mice were euthanized via CO_2_ asphyxiation followed by cervical dislocation prior to muscle collection.

### 2.3 Plasmid DNA isolation

Plasmid DNA was isolated from glycerol stocks of E. coli containing TurboGFP shMIMIC Mouse Lentiviral miRNA control or miR‐1a‐3p plasmids (Dharmacon), according to the manufacturer's instructions. Briefly, E. coli containing the target plasmids was incubated in LB broth medium with 100 μg/mL carbenicillin for 18 h at 37°C. Plasmid DNA was isolated using the QIAGEN Plasmid Mini Kit (# 12123 and 12125), according to the manufacturer's instructions.

### 2.4 HEK293T culture

HEK293T (ATCC Number CRL‐3216) cells were maintained in a medium composed of high‐glucose Dulbecco's modified Eagle medium (DMEM) supplemented with 10% fetal bovine serum (FBS), 100 units/mL penicillin, 100 μg/mL streptomycin., and 6 mM L‐glutamine. For lentivirus production, the medium was replaced with reduced serum medium containing high‐glucose DMEM, 5%FBS, 2 mM L‐glutamine, 100 units/mL of penicillin, and 100 μg/mL streptomycin.

### 2.5 Lentivirus production

HEK293T cells were used to produce the lentiviral vectors. Cells (1.2 × 10^6^) were seeded at 70%–80% confluency in a 6‐well plate and transfected with 1 μg of the target plasmid DNA (control or miR‐1‐mimic) using the DharmaconTM Trans‐Lentiviral packaging kit according to the manufacturer's instructions. After 16 h of transfection, cells were incubated with reduced serum medium and transfection efficiency was calculated based on the number of cells containing the TurboGFP reporter gene. After 48 h, the virus‐containing media were collected, spanned down for pelleting cell debris, and filtered through a 0.45 μm sterile filter (Fisher). Filtered supernatants were concentrated by ultracentrifugation at 95,300 × *g* in Rotor SW60 Ti for 2 h at 4°C. The pellet‐containing viral particles were resuspended in 1 mL of DMEM. A final low‐speed centrifugation (25,000 × *g* for 10 min at 4°C) was performed to remove serum.

### 2.6 In vivo miRNA mimic treatment

During MOV or sham surgeries, mice received 50 μL (1 × 10^8^ IU/mL) of control (left plantaris) and mimic (right plantaris) lentiviral particles distributed over two injections, one proximal and one distal, using an insulin syringe (BD). Plantaris muscles were collected after 10 days.

### 2.7 RNA isolation and cDNA synthesis

Total RNA was isolated from the plantaris muscle samples using TRIzol Reagent (15596026, Life Technologies). Frozen muscle samples were homogenized using beads and the Bullet Blender Gold CE (Next Advance, Troy, NY, USA). Following homogenization, RNA was isolated via phase separation by addition of bromochloropropane. The aqueous phase was transferred to a new tube and further processed on columns using the Direct‐zol Kit (R2072, Zymo Research). RNA was treated in‐column with DNase and eluted in nuclease‐free water. RNA concentration and purity were assessed using a Nanodrop 2000 spectrophotometer (ThermoFisher Scientific). Reverse transcription reactions for miR‐1 and U6 small nuclear RNA (Rnu6) were performed with 10 ng of total RNA using Taqman MicroRNA Reverse Transcription Kit (4366596, ThermoFisher Scientific) according to the manufacturer's instructions. For mRNA, quantitative PCR (qPCR) 500 ng of total RNA was reverse transcribed using SuperScript™ IV VILO™ Master Mix (11756050, Invitrogen) according to the manufacturer's instructions.

### 2.8 mRNA and miRNA expression

For mRNA, quantitative PCR (qPCR) was run using iTaq Universal SYBR Green Supermix (1725120, Bio‐Rad). For miRNA, Taqman Universal PCR Master Mix (2x) (4304437, ThermoFisher Scientific) and TaqMan gene expression assay (miR‐1, #002222; Rnu6, #001973) were used for qPCR. qPCR was performed using the CFX Connect Real‐Time PCR Detection System as described by the manufacturer. qPCR efficiency was calculated by linear regression during the exponential phase using the LinRegPCR software v11.1 (Vechetti et al., 2021). miR‐1 and mRNAs (Itgb1bp2, Itm2a) levels among the groups were compared following normalization with Rnu6 (for miRNAs) and VCP (for mRNA) (most stable among groups). Primer sequences are described in Table 1.

**TABLE 1:** RT‐PCR primers utilized.

Primer target	Sequence (5′ – >3′)
Itm2a‐F	CAAGATGTAGAGGCGCTCGT
Itm2a‐R	TTTCTCCTGCGGGACAACTC
Itgb1bp2‐F	ACCAACGCTTCCATGTCTCT
Itgb1bp2‐R	GAGGCAACTCTGACCAGGAAG
Vcp‐F	CTCCCTCCAAAGGCGTTCTT
Vcp‐R	TGGCCTCAGATTCCCCAAAC

### 2.9 RNA‐seq

Libraries (*n* = 5/group) were prepared using the NEBNext Ultra II RNA Library Prep Kit, and the resulting cDNA was checked with Qubit and real‐time PCR for quantification and a bioanalyzer for size distribution detection. The quantified libraries were pooled and sequenced on the Illumina platform. Quality control preprocessing was performed with FastQC v0.11.9, followed by adapter and low‐quality base removal using Cutadapt 4.4 and Trim Galore 0.6.10, respectively. Low quality base calls (Phred score < 33) were removed prior to trimming adapter sequences. The reads were aligned to the GRCm38reference genome using Hisat2 v2.0.5. FeatureCounts v1.5.0‐p3 was used to count the number of reads mapped to each gene. Raw counts from RNA‐seq were used as inputs into R (Version 4.1.0). After filtering the low‐expression genes, DESeq2 (Version 1.34.0) was used for normalization and differential analyses of RNA‐seq data to identify differentially expressed genes (DEGs) between groups. DEGs were identified using a false discovery rate (Benjamini–Hochberg method) with an adjusted *p* value <0.05. DEGs with a log2 fold change (Log2FC) over 1 and lower than −1 and adj. *p* < 0.05 were used for downstream functional analysis. Gene Ontology (GO) enrichment analysis of differentially expressed genes was implemented by the clusterProfiler R package, in which gene length bias was corrected. GO terms with an adjusted *p* value <0.05 were considered significantly enriched. We used all the genes detected in our sequencing as the background for the GO analysis.

### 2.10 eCLIP‐sequencing

For the chimeric CLIP‐sequencing, we used adult 5‐month‐old male plantaris muscles from control and miR‐1 mimic treated mice that underwent 10 days of MOV (*n* = 1). Muscle samples were snap‐frozen in liquid nitrogen, and chimeric CLIP‐sequencing was performed by Eclipse Bioinnovations, Inc. (Eclipse Bioinnovations). Approximately 12 mg of mouse plantaris muscle was homogenized by cryogenic pulverization. The samples were then UV (254 nm) cross‐linked twice at 400 mJ/cm^2^ using Stratalinker 2400 (Stratagene) on a bed of ice. After cross‐linking, the samples were sonicated (QSonica Q800R2; QSonica LLC) to shear genomic DNA into smaller fragments. Ago2 immunoprecipitation using an Ago2 antibody (50683‐RP02, SinoBiological) was pre‐coupled to anti‐mouse Dynabeads (M‐280 Sheep Anti‐Mouse IgG Dynabeads, 11201D; Thermo Fisher Scientific), added to the homogenized lysate, and incubated overnight at 4°C with gentle rocking. After immunoprecipitation, 2% of the samples were used as paired input samples. For chimeric eCLIP experiments, a standardized eCLIP protocol (Van Nostrand et al., 2016) was modified to enable chimeric ligation of miRNAs and mRNA (Manakov et al., 2022). miRNA‐specific chimeric eCLIP was performed by amplifying cDNA using a mouse miR‐1 primer and a sequencing adapter‐specific primer after amplification with indexed primers.

Read processing and cluster analysis of miR‐1 eCLIP were performed as previously described (Van Nostrand et al., 2016; Vogler et al., 2018). Briefly, the 3′ barcodes and adapter sequences were removed using standard eCLIP scripts. The reads were trimmed, filtered for repetitive elements, and aligned to the mm9 reference sequence using STAR. PCR duplicate reads were removed based on the read start positions and random sequence. Bigwig files for the genome browser display were generated based on the location of the second paired‐end read. Peaks were identified using the encode_branch version of CLIPPER using the parameter “‐smm9.” Peaks were normalized against size‐matched input by calculating fold enrichment of reads in IP versus input and were designated as significant if the number of reads in the IP sample was greater than that in the input sample, with a Bonferroni corrected Fisher's exact *p* value <10^−8^.

### 2.11 Immunohistochemistry

Plantaris muscles were removed and pinned to a cork block at resting length, covered with a thin layer of Tissue Tek optimal cutting temperature (OCT) compound (Sakura Finetek, Torrance, CA), quickly frozen in liquid nitrogen‐cooled isopentane, and stored at −80°C until sectioning. Frozen muscles were sectioned (7 μm), air‐dried for at least an hour and stored at −20°C. For GFP detection, slides were fixed in 4% paraformaldehyde, and then sections were blocked for 1 h with 2.5% normal horse serum (Vector Laboratories, #S‐2012) and Mouse‐on‐Mouse Blocking Reagent (Vector Laboratories, #MKB‐2213). To address the potential issue of GFP fluorescence reduction or loss due to freezing processing steps, we elected to use GFP antibody for reliable detection. The sections were then incubated with anti‐dystrophin and anti‐GFP (1:100, Abcam, Cambridge, MA, #ab13970) overnight at 4°C. The sections were washed with PBS and incubated with species‐specific secondary antibodies (1:250, Life Technologies, #A‐11001 and A‐21467). The sections were subsequently washed with PBS and co‐stained with DAPI prior to mounting with anti‐fade media. For dystrophin identification, muscle sections were rehydrated with PBS and blocked with a mouse‐on‐mouse blocking reagent (Vector Laboratories). After washing, the slides were incubated with an anti‐dystrophin antibody (1:50; cat. no. VPD505; Vector Laboratories) overnight, was followed by incubation for 75 min with goat anti‐mouse biotinylated secondary antibody (1:1000, 115‐065‐205; Jackson ImmunoResearch). The sections were washed again, incubated for 30 min with SA‐FITC (1:150, no. SA‐5001; Vector Laboratories) and postfixed in 4% paraformaldehyde before mounting using Vectashield fluorescent mounting medium with DAPI (Vector Laboratories). All images were acquired using an upright microscope at ×20 magnification (AxioImager M1, Zen 2.3 Imaging Software; Zeiss). The entire muscle cross‐sections were captured, and the muscle fiber cross‐sectional area for all the fiber (GFP positive and negative fibers) was determined using MyoVision software developed by Wen et al. (2018).

### 2.12 Statistical analysis

Unless otherwise stated, all statistical comparisons of multiple variables (e.g., time and genetic cohorts) were performed using two‐way ANOVA. All ANOVA tests were corrected using Tukey HSD test. Values of significance are stated in the figure legends. The number of replicates used is directly stated in the figure legend or the Materials and Methods section. Real‐time quantitative PCR validation statistical analyses were performed using repeated measures two‐way ANOVA to determine significance, and the results are presented as mean ± SD. Differences were considered statistically significant at *p* ≤ 0.05. All statistical tests (except for sequencing data) were performed using GraphPad Prism version 9.

### 2.13 Resource availability

Both RNA‐seq and eCLIP‐seq raw data were deposited in the NCBI Gene Expression Omnibus database (GSE265982 and GSE265946).

## 3 RESULTS

### 3.1 Blockage of muscle‐specific miR‐1 downregulation blunts muscle hypertrophy following MOV

**FIGURE 1:**
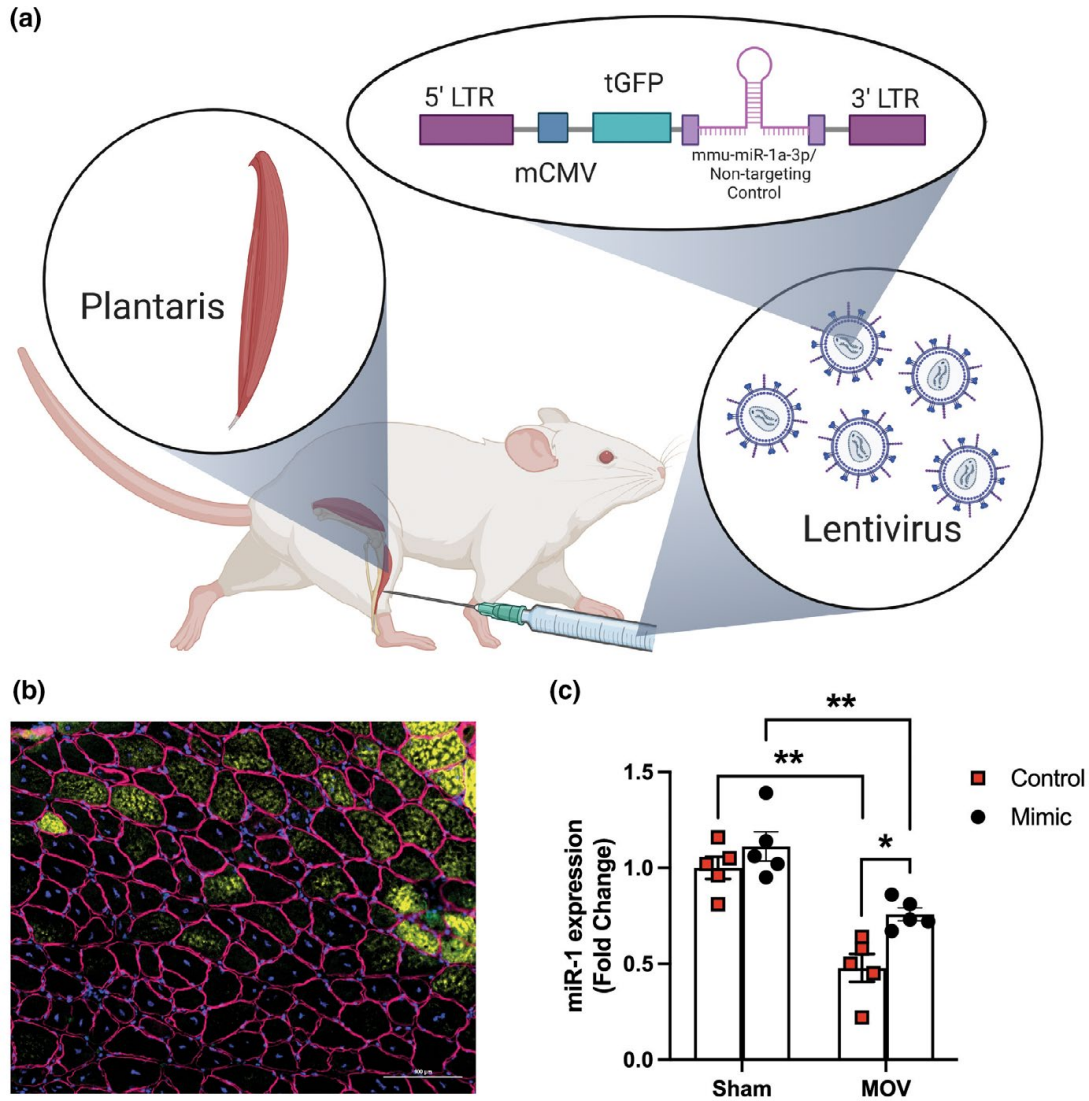
Efficiency of mir‐1 mimic lentivirus. (a) The mir‐1 mimic and control lentivirus design sequences are shown, along with the injection location. (b) IHC images illustrate the expression of GFP in muscle fibers, with increased expression observed in the mimic compared to the control. These results show that miR‐1 mimic lentivirus successfully mediated the expression of miR‐1 in plantaris muscle. (c) Total relative miR‐1 expression levels between the Sham‐ or mechanical overload (MOV)‐surgery after mimic or control virus treatments are presented, with a significant increase in miR‐1 mimic observed in the MOV. *p* value with statistical significance (*p* < 0.05) are displayed. Two‐Way Repeated Measures ANOVA followed by Tukey's multiple comparisons test. *p < 0.05 and **p < 0.01.

To mechanistically test the role of miR‐1 in skeletal muscle hypertrophy, we injected, in a contralateral approach, lentivirus‐containing either scramble‐control (left plantaris) or miR‐1‐mimic (right plantaris) under a mouse CMV promoter and Turbo‐GFP as a reporter gene during sham or synergist ablation surgeries (Figure 1a). To assess whether our strategy successfully increased miR‐1 levels, we performed immunohistochemistry for GFP and qPCR for miR‐1 (Figure 1b,c). As shown in Figure 1b, we observed an average of 55% GFP‐positive fibers within the dystrophin border, indicating successful infection with the lentivirus in the muscle fibers. In addition, we observed a smaller decrease in miR‐1 levels (25%) in mimic‐injected plantaris compared to a 50% reduction in the control after 10 days of MOV‐induced muscle hypertrophy (Figure 1c). Intriguingly, we did not observe an increase in miR‐1 levels in the plantaris of the sham group after the mimic injection (Figure 1c), despite observing the same GFP intensity as in the MOV group. Although speculative, we believe that the additional copies of miR‐1, the most abundant miRNA in the skeletal muscle (Vechetti et al., 2021), via mimic injection stimulated miR‐1 turnover in the sham group, preventing its accumulation.

**FIGURE 2:**
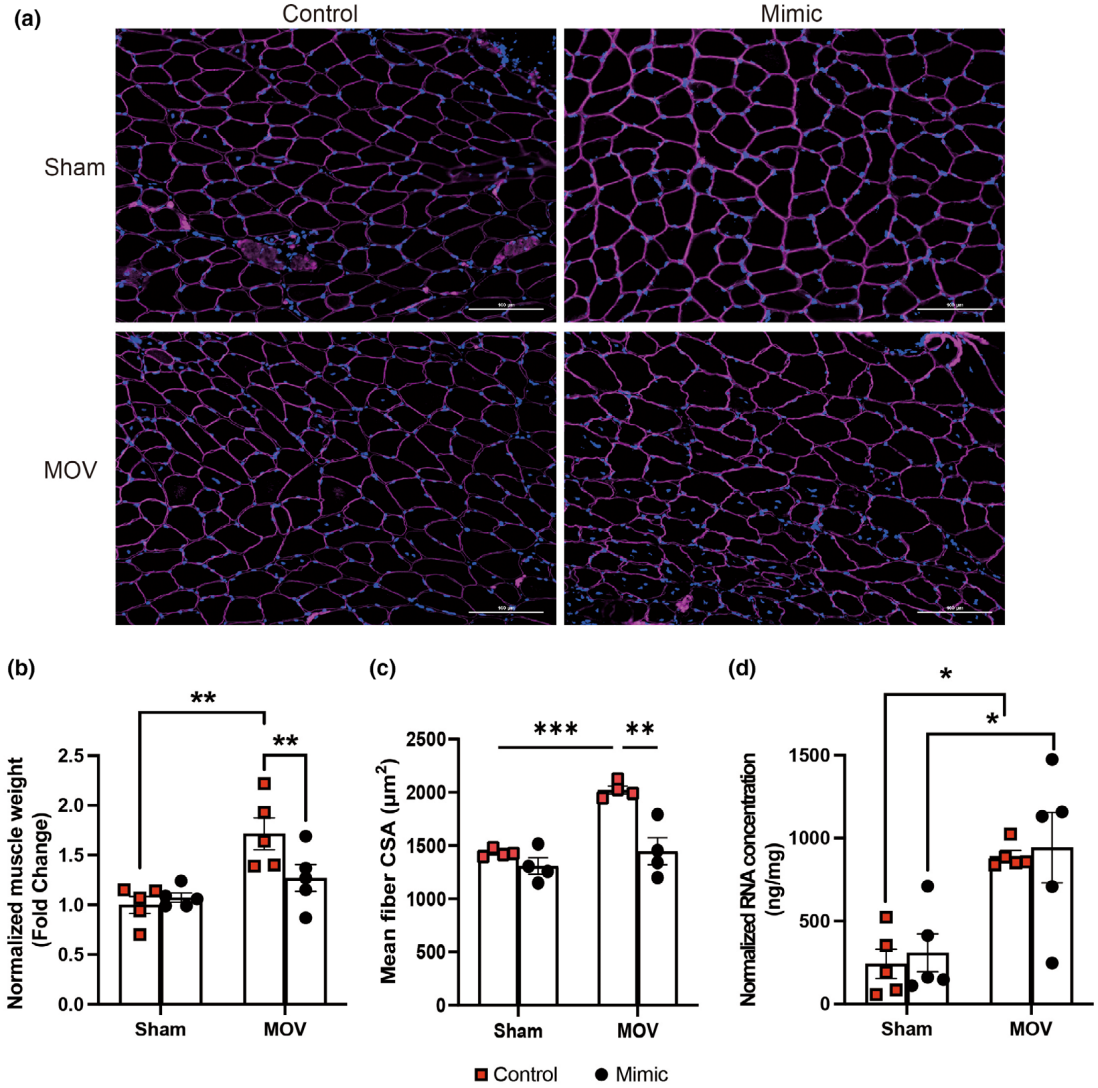
The effects of mir‐1 on muscle hypertrophy. (a) are representative IHC images displaying dystrophin (purple) and myonuclei (DAPI). (b) shows muscle weight normalized by body weight between sham and mechanical overload (MOV) in control‐ or mimic administrated plantaris. (c) compares the muscle fiber cross‐sectional area between groups. (d) displays the total RNA concentration relative to muscle weight. *p < 0.05, **p < 0.01, and ***p < 0.001 displayed statistical significance. Two‐Way Repeated Measures ANOVA followed by Tukey’s multiple comparisons test.

Despite not completely blocking miR‐1 reduction after MOV‐induced muscle hypertrophy, the smaller reduction (25% vs. 50% in the control) observed in the mimic‐injected plantaris was sufficient to blunt muscle hypertrophy, as assessed by normalized muscle weight (Figure 2b), and muscle cross‐sectional area (Figure 2a,c). Interestingly and contrary to our hypothesis, the total RNA concentration following 10 days of MOV‐induced muscle hypertrophy was not affected in the plantaris‐injected mimic (Figure 2d) despite a blunted growth response, indicating that ribosome biogenesis was not regulated by miR‐1 levels. Control‐injected plantaris showed a normal hypertrophic response to MOV, with an increase in normalized muscle weight, CSA, and total RNA concentration (Figure 2a–d). Finally, we observed an increase in centrally located nuclei in mice that underwent MOV compared to sham, irrespective of miR‐1 manipulation (0.51 MOV CTL vs. 0.57 MOV Mimic central nuclei/fiber). These findings suggest the possibility of subtle muscle damage and/or regeneration resulting from lentivirus injection during MOV‐induced hypertrophy.

### 3.2 Potential mechanisms by which miR‐1 regulates MOV‐induced muscle hypertrophy

**FIGURE 3:**
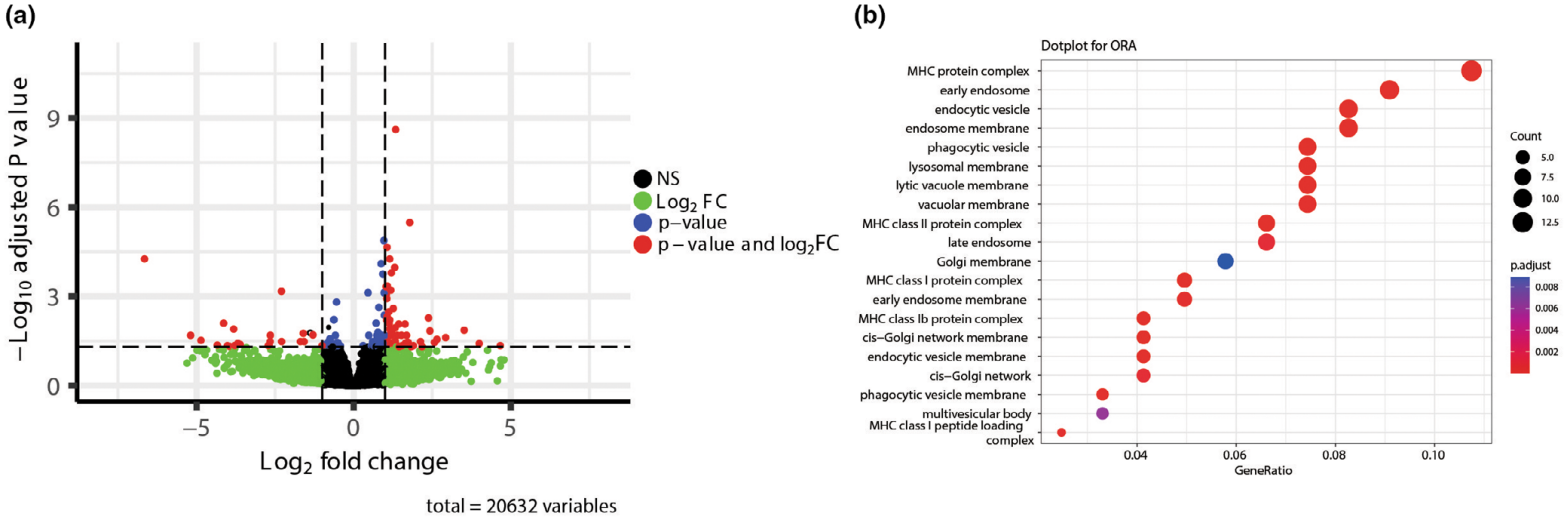
RNA‐seq analysis of differentially expressed genes. (a) shows a volcano plot of significantly different genes displaying the log2 fold change on the x‐axis and the negative log10 of the adjusted *p* value on the y‐axis. The left side dots represent downregulated genes in the control‐administrated plantaris, while the right‐side dots represent upregulated genes. The significantly differentially expressed genes after adjusted *p* value are labeled in red. (b) shows a dot plot displaying the significant difference GO pathways identified from the RNA‐seq analysis. The size of the dots reflects the number of genes in each GO term, and the color represents the adjusted *p* value.

To understand how preventing greater miR‐1 reduction during MOV blunts muscle hypertrophy without affecting the increase in total RNA concentration, we performed RNA‐seq in the plantaris‐injected control and mimic between sham and MOV‐induced muscle hypertrophy. As expected, based on our inability to increase miR‐1 levels in the sham group, we did not observe any significant differences in gene expression between the mimic and control groups. Ten days after MOV‐induced muscle hypertrophy, 137 differentially expressed genes (DEGs) were identified in the mimic compared to the control, of which 22 were downregulated and 52 were upregulated (Figure 3a and Table S1). Gene ontology (GO) enrichment analysis revealed that the most enriched biological pathways were related to membrane‐related processes (Figure 3b).

**FIGURE 4:**
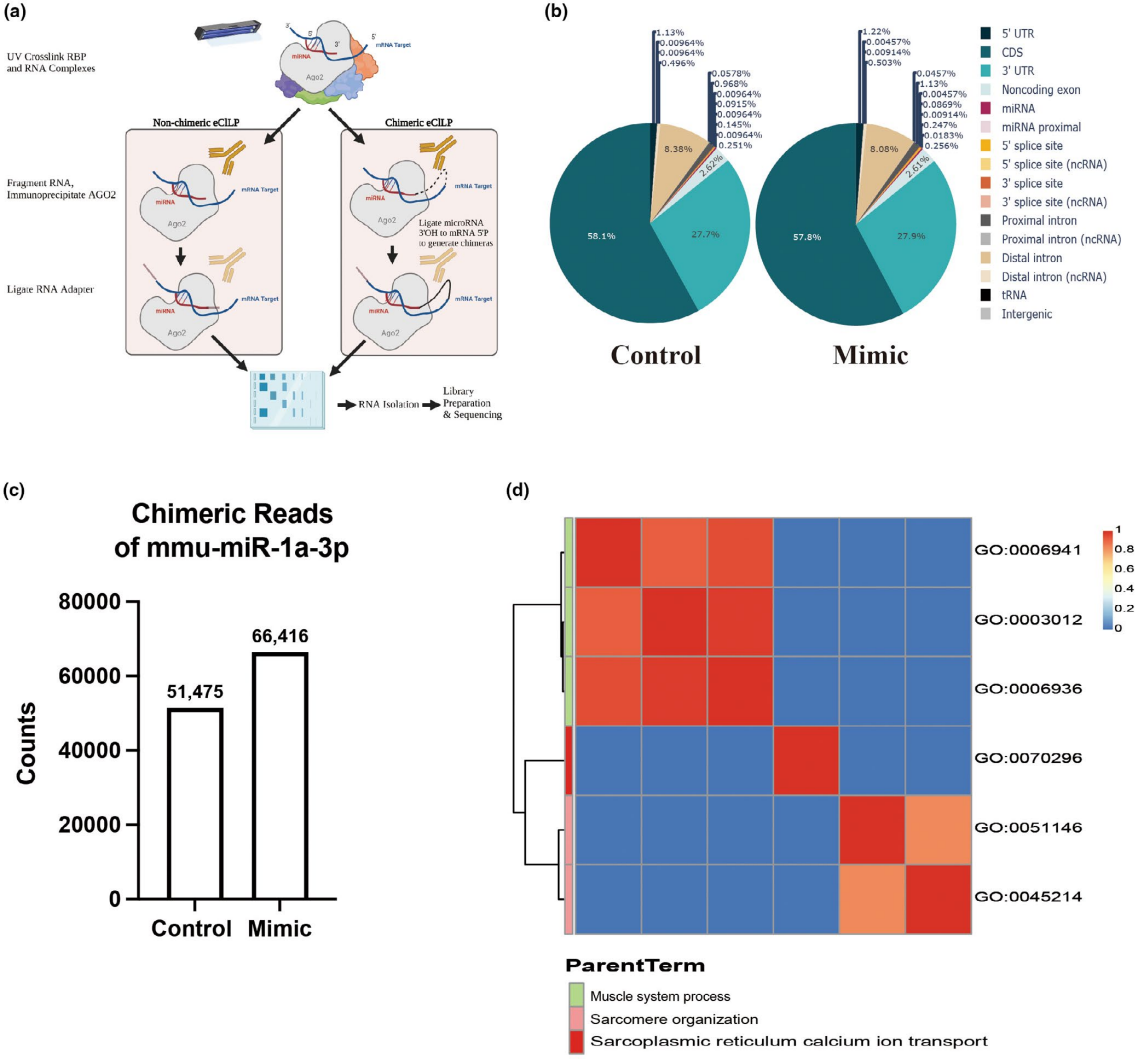
eCLIP‐seq analysis of microRNA targets. (a) depict a standard eCLIP workflow. (b) pie chart displaying the position of microRNA targets on mRNA between groups. The different colors represent different positions of the target site. (c) shows a bar graph displaying the chimeric reads of miR‐1 between groups. (d) presents a heatmap illustrating the correlation between target genes uncovered through eCLIP‐seq and various biological processes.

Since we did not observe any differences in the sham group, we focused our analyses on the MOV‐induced muscle hypertrophy groups. To identify potential targets of miR‐1 that could explain the blunted hypertrophic effect observed in our study, we performed exploratory (*n* = 1) eCLIP‐seq analysis of mice that had undergone 10 days of MOV‐induced muscle hypertrophy (Figure 4a). First, we analyzed potential differences in the target sites between the control and mimic‐injected plantaris. We did not observe differences in the target sites between treatments; most of these miRNA target sites were located in the mRNA coding sequence (CDS), followed by the 3′UTR (Figure 4b). As shown in Figure 4c, miR‐1 abundance was 30% higher in the mimic‐injected plantaris than in the control‐injected plantaris, which is consistent with our qPCR results (Figure 2b). In addition, most of the target genes identified in our exploratory eCLIP‐seq analysis in the CDS and 3′UTR were associated with skeletal muscle organization (Figure 4d) shows the association between target genes identified from eCLIP‐seq and different biological process.

**FIGURE 5:**
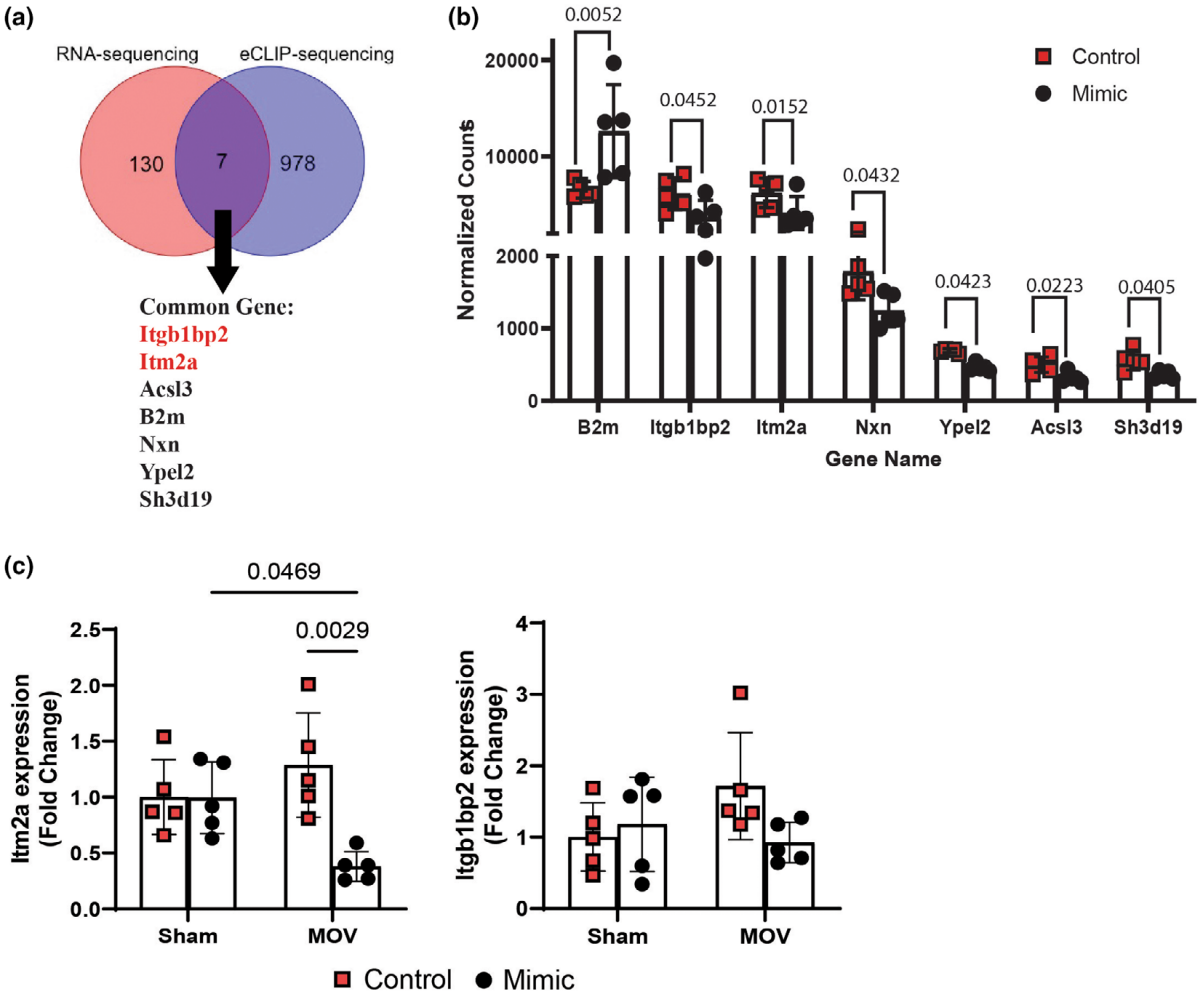
Integration of RNA‐seq and eCLIP‐seq results. (a) shows a Venn diagram of the significantly different genes identified from RNA‐seq and the miR‐1 targeted genes from eCLIP‐seq. The names of the seven common genes are shown under the overlapping region. (b) bar plot showing the normalized counts of these seven genes on MOV between control and mimic from RNA‐seq results. (c) qPCR validation of *Itm2a* and *Itgb1bp2 expression*. P value with statistical significance (*p* < 0.05) are displayed. Two‐Way Repeated Measures ANOVA followed by Tukey's multiple comparisons test.

Next, to identify potential targets regulated by miR‐1, we integrated the DEGs identified in RNA‐seq analysis with the identified miR‐1 target genes (985 genes) in the eCLIP‐seq dataset (Appendix S1). This comparison resulted in only seven genes that were differentially expressed and targeted by miR‐1 (Figure 5a). Of the seven identified genes, only ß2 microglobulin (*B2m*) was upregulated whereas the other six genes were downregulated in the mimic group. Specifically, we identified *Itgb1bp2*, *Itm2a*, Nucleoredoxin (*Nxn*), Yippee Like 2 (*Ypel2*), Acyl‐CoA Synthetase Long Chain Family Member 3 (*Acsl3*), and SH3 Domain Containing 19 (*Sh3d19*) (Figure 5b). Finally, in order to validate our integration analysis, we performed qPCR for *Itgb1bp2*, *Itm2a*, and membrane‐related proteins. As observed in Figure 5c, our results suggest that *Itm2a* expression is affected by miR‐1 regulation, confirming its potential involvement with skeletal muscle hypertrophy. Although, *Itgb1bp2* expression followed the same trend, we did not observe statistical differences.

## 4 DISCUSSION

Skeletal muscle hypertrophy is a complex process that involves coordination of a cascade of signaling pathways, epigenetic modifications, satellite cells, miRNA regulation, and an increase in muscle fiber size. With regard to miRNAs, miR‐1 has attracted particular interest because its dynamic regulation during skeletal muscle hypertrophy suggests an important role in this process (Chaillou et al., 2013; Drummond et al., 2008; D'Souza et al., 2019; McCarthy & Esser, 2007; Vechetti et al., 2019, 2021). Here, by preventing the robust reduction in miR‐1 levels during MOV‐induced muscle hypertrophy, we confirmed miR‐1 involvement in muscle hypertrophy and identified membrane‐associated proteins as potential new targets for the regulation of skeletal muscle mass.

miRNAs have been shown to fine‐tune gene expression either by accelerating the degradation of mRNA and/or by inhibiting translation in a diverse range of biological processes, including development, differentiation, homeostasis, and diseases (Bartel, 2009; Sayed & Abdellatif, 2011). The identification of myomiRs (McCarthy, 2008; Sempere et al., 2004) has expanded our knowledge of the molecular network within skeletal muscles. The pioneering study by McCarthy and Esser demonstrated a reduction in myomiRs, miR‐1, and ‐133a, during MOV‐induced muscle hypertrophy, paving the way for other studies demonstrating the potential role of miRNAs in the regulation of skeletal muscle mass (Chaillou et al., 2013; Clop et al., 2006; Davidsen et al., 2011; Drummond et al., 2008; D'Souza et al., 2019; Fry et al., 2017; Hitachi et al., 2014; Ismaeel et al., 2024; Lim et al., 2023; Motohashi et al., 2013; Murach, Mobley, et al., 2020; Murach, Vechetti Jr, et al., 2020; Vechetti et al., 2019, 2021).

Although there is an abundance of evidence suggesting the importance of miRNAs in the regulation of muscle mass, several studies have utilized only correlations (i.e., decreasing miRNA expression and increasing muscle mass/potential target) to validate or refute this hypothesis. In our study, we reasoned that preventing/decreasing the degree of miR‐1 downregulation during MOV‐induced muscle hypertrophy would allow us to rigorously test the idea of miR‐1 acting as a “molecular brake” to muscle growth. Here, we show that miR‐1 downregulation is necessary for skeletal muscle growth after 10 days of MOV‐induced muscle hypertrophy. Interestingly, the blunted hypertrophy observed in the plantaris‐injected mimic group was not associated with a decrease in total RNA concentration, which is a readout of ribosome biogenesis. Indeed, several studies have shown that ribosome biogenesis is necessary for skeletal muscle hypertrophy (Figueiredo & McCarthy, 2019; Kim et al., 2019; Kirby et al., 2016; von Walden et al., 2012; Wen et al., 2016), and some studies have demonstrated that miRNAs, including miR‐1 (Sun et al., 2023), regulate the steps of ribosome biogenesis (Bryant et al., 2024; McCool et al., 2020).

To gain insight into the mechanisms by which reduction of miR‐1 downregulation regulates muscle hypertrophy, we first performed RNA sequencing and observed a small change (137 DEGs) between the plantaris‐injected control and mimic after 10 days of MOV‐induced muscle hypertrophy. The most significantly enriched pathways related to the DEGs were associated with membrane‐related processes. Although unexpected, these findings are in line with previous studies that have reported the involvement of membrane‐associated proteins in muscle hypertrophy (Flück et al., 1999; Hornberger et al., 2006; O'Neil et al., 2009; You et al., 2012, 2014). For example, Chaillou et al. (2013) demonstrated that the integrin‐linked kinase pathway is upregulated during MOV‐induced muscle hypertrophy. Despite the changes observed in mice submitted to MOV, we did not identify any DEGs between the sham groups, which reflects our inability to stimulate an increase in miR‐1 expression in these groups.

Finally, to identify miR‐1‐targeted genes, we integrated our RNA‐seq and eCLIP‐seq datasets and identified seven genes (*B2m*, *Itgb1bp2*, *Itm2a*, *Nxn*, *Yepl*, *Acsl3*, and *Sh3d19*) that were both differentially expressed and miR‐1 targets. Since the expression of *B2m*, a component of MHC class I molecules, was elevated after MOV‐induced muscle hypertrophy, we focused our attention on the other six genes. Nxn is a member of the thioredoxin family of proteins that is involved in various biological processes by regulating oxidative stress (Funato & Miki, 2007). Although its role in skeletal muscle hypertrophy has not been explored, it has been reported to regulate the Wnt/beta‐catenin pathway (Funato et al., 2010), which can induce skeletal muscle hypertrophy (Armstrong & Esser, 2005). We were unable to identify studies relating *Ypel2* to skeletal muscle hypertrophy, but a member of this family, *Ypel4*, has been shown to be essential for red blood cell membrane integrity (Mattebo et al., 2021), suggesting that perhaps *Ypel2* could also regulate cell membrane. The genes *Acsl3* and *Sh3d19* have also not been linked to muscle hypertrophy, but they are involved in fatty acid flux and glucose uptake in myotubes (Jung & Bu, 2020) and the regulation of metalloproteases and cytoskeletal organization (Tanaka et al., 2004), respectively.

Finally, both *Itm2a* and *Itgb1bp2* are membrane‐associated proteins, supporting our RNA‐seq results that membrane‐related pathways play a crucial role in muscle hypertrophy, which seems to be regulated by miR‐1 downregulation. *Itm2a* encodes a type II membrane protein that belongs to integral membrane proteins and has been shown to be a positive regulator of autophagy through an mTOR‐dependent manner (Zhou et al., 2019). To the best of our knowledge, there are only a handful of studies investigating the role of *Itm2a* in skeletal muscle. These studies demonstrated *Itm2a* to have an important role on muscle fiber formation and differentiation, and perhaps regeneration through its interaction with Pax3/Pax7 (Lagha et al., 2013; Van den Plas & Merregaert, 2004). On the contrary, *Itgb1bp2* (also known as Melusin) is expressed specifically in skeletal and heart muscles. While its role in muscle hypertrophy has not been investigated, melusin functions by binding to the cytoplasmic domain of β1 integrin, which acts as a membrane receptor that anchors sarcomeres to the plasma membrane (Brancaccio et al., 1999). Interestingly, MOV‐induced muscle hypertrophy has been shown to induce an increase in the integrin‐linked kinase (ILK) pathway, suggesting that transmembrane integrins may signal an upregulation in other genes that coordinate the anabolic response (Chaillou et al., 2013). In addition, mice overexpressing the α(7)BX2‐integrin in skeletal muscle sensitizes skeletal muscle to mechanical strain and subsequent growth, which seems to be uncoupled from mTORC1 activation (Boppart et al., 2006; Boppart & Mahmassani, 2019; Zou et al., 2011).

While our results do not elucidate the mechanism by which miR‐1 regulates MOV‐induced muscle hypertrophy, they provide additional evidence indicating that membrane‐related proteins play a significant role in the regulation of skeletal muscle mass. Our study has several limitations. Firstly, our eCLIP‐seq analysis was restricted to a single sample per group, potentially affecting the overall reliability of our results. Additionally, our findings are based solely on gene expression and male mice, and the absence of protein‐level validation and female mice constrains our discussion to a more speculative interpretation. Another notable limitation of our study is the observation of a significant increase in centrally located nuclei only in mice that underwent MOV‐induced muscle hypertrophy, irrespective of miR‐1 manipulation (i.e., scramble vs. mimic). While these results suggest that the accumulation of centrally located nuclei is likely an adverse effect of lentivirus administration during the MOV procedure, we cannot definitively exclude the possibility of subtle muscle damage or regeneration influencing our results. Unfortunately, due to tissue limitations and resource constraints, we were unable to perform follow‐up and validation studies. Future investigations should incorporate detailed histological analysis and validation of the membrane‐related genes to more comprehensively examine the effects of miR‐1 overexpression on muscle integrity and regeneration. Lastly, we observed that the use of the miR‐1 mimic virus significantly upregulated the expression of miR‐1 in the MOV‐induced muscle hypertrophy but not in the sham groups. This may be attributed to the fact that skeletal muscle cells already contain high levels of miR‐1, and the delivery of additional copies triggered the turnover of excess miR‐1. However, further experimentation is necessary to confirm this hypothesis. Furthermore, the use of CMV as a promoter can impact non‐muscle cells, which might have influenced he final results. Despite these limitations, by integrating CLIP‐seq with RNA‐seq data, we were able to gain valuable insights into the role of miR‐1 in muscle hypertrophy.

In conclusion, this study demonstrates that miR‐1 downregulation is necessary for muscle hypertrophy and suggests the potential significance of membrane‐associated gene such as Itm2a in this process. These findings provide a foundation for future research to corroborate or refute our results through validation of the targets identified in this study and investigation of the functional role of these targets in muscle hypertrophy.

## AUTHOR CONTRIBUTIONS

S.F. and B.D.R. performed multiple experiments throughout. J.S.G., C.B.M., M.D.R., and F.v.W. conducted immunohistochemistry experiments and/or contributed substantial resources to complete the study. S.F. and I.J.V. were primarily responsible for drafting the manuscript, and all co‐authors provided feedback and intellectual input. All authors have reviewed and approved the final version of this manuscript.

## ETHICS STATEMENT

This study was approved by University of Nebraska Institutional Animal Care and Use Committee (approval #2162).

## Supporting information


Table S1.

Appendix S1.

## Data Availability

The datasets presented in this study can be found in online repositories through the GSE numbers provided in the manuscript.
