# Supplementary material for: miRNA‐1 regulation is necessary for mechanical overload‐induced muscle hypertrophy in male mice
Source: Physiol Rep. 2025 Jan 6;13(1):e70166. doi: 10.14814/phy2.70166 (PMC11705529; doi:10.14814/phy2.70166)
Supplement: Supplementary file 1 — Table S1. [file PHY2-13-e70166-s001.docx]

| **Supplemental Table 1.** Differentially expressed genes after 10 days of mechanical overload between mimic compared to the control | | | | |
| --- | --- | --- | --- | --- |
| **ID** | **Symbol** | **log2FoldChange** | **pvalue** | **padj** |
| ENSMUSG00000077493 | Snord91a | -6.6554 | 1.94E-08 | 5.50E-05 |
| ENSMUSG00000115976 | Gm31462 | -5.18823 | 6.94E-05 | 0.02061 |
| ENSMUSG00000087196 | Gm13373 | -4.85765 | 0.000123 | 0.030437 |
| ENSMUSG00000053168 | 9030619P08Rik | -4.33796 | 0.000269 | 0.044089 |
| ENSMUSG00000043999 | Gpr75 | -4.13752 | 1.55E-05 | 0.008036 |
| ENSMUSG00000040569 | Slc26a7 | -4.01708 | 0.000325 | 0.045986 |
| ENSMUSG00000097303 | 3110083C13Rik | -3.95686 | 0.000358 | 0.04796 |
| ENSMUSG00000033805 | Ephx4 | -3.81762 | 2.78E-05 | 0.012689 |
| ENSMUSG00000087644 | Gm14703 | -3.78729 | 0.000297 | 0.045621 |
| ENSMUSG00000000049 | Apoh | -3.68483 | 0.000215 | 0.038683 |
| ENSMUSG00000046683 | 0610025J13Rik | -3.60942 | 0.000242 | 0.042005 |
| ENSMUSG00000048782 | Insc | -2.71744 | 0.000299 | 0.045621 |
| ENSMUSG00000023043 | Krt18 | -2.69013 | 0.00028 | 0.044949 |
| ENSMUSG00000083820 | Ndufs6b | -2.65923 | 0.000152 | 0.033502 |
| ENSMUSG00000037463 | Fbxo27 | -2.65246 | 5.79E-05 | 0.020511 |
| ENSMUSG00000064307 | Lrrc51 | -2.29735 | 5.59E-07 | 0.00068 |
| ENSMUSG00000071037 | Camkmt | -2.29227 | 0.000149 | 0.033502 |
| ENSMUSG00000052565 | H1f3 | -1.6946 | 0.000158 | 0.033502 |
| ENSMUSG00000054871 | Tmem158 | -1.60324 | 4.46E-05 | 0.017654 |
| ENSMUSG00000039684 | Gm5422 | -1.57544 | 0.00015 | 0.033502 |
| ENSMUSG00000111375 | Btbd8 | -1.29625 | 5.27E-05 | 0.019955 |
| ENSMUSG00000010751 | Tnfrsf22 | -1.00781 | 0.000316 | 0.045986 |
| ENSMUSG00000113101 | Gm33424 | 4.664777 | 0.000324 | 0.045986 |
| ENSMUSG00000015619 | Gata3 | 3.995379 | 0.000223 | 0.039073 |
| ENSMUSG00000044162 | Tnip3 | 3.516122 | 3.17E-05 | 0.013832 |
| ENSMUSG00000097194 | 9330175E14Rik | 2.927097 | 9.22E-05 | 0.024511 |
| ENSMUSG00000044309 | Apol7c | 2.65124 | 0.000112 | 0.028066 |
| ENSMUSG00000024910 | Ctsw | 2.571601 | 0.000179 | 0.035578 |
| ENSMUSG00000037548 | H2-DMb2 | 2.425293 | 3.43E-05 | 0.014595 |
| ENSMUSG00000025279 | Dnase1l3 | 2.381283 | 8.17E-06 | 0.005351 |
| ENSMUSG00000005947 | Itgae | 2.174462 | 0.000392 | 0.049817 |
| ENSMUSG00000051998 | Lax1 | 2.126443 | 0.000157 | 0.033502 |
| ENSMUSG00000045322 | Tlr9 | 1.898273 | 0.000327 | 0.045986 |
| ENSMUSG00000076498 | Trbc2 | 1.830338 | 0.000374 | 0.049715 |
| ENSMUSG00000027514 | Zbp1 | 1.784109 | 3.87E-10 | 3.30E-06 |
| ENSMUSG00000020399 | Havcr2 | 1.733125 | 0.00018 | 0.035578 |
| ENSMUSG00000028337 | Coro2a | 1.710223 | 0.000178 | 0.035578 |
| ENSMUSG00000038179 | Slamf7 | 1.683095 | 7.22E-05 | 0.02061 |
| ENSMUSG00000022636 | Alcam | 1.646279 | 0.000201 | 0.038072 |
| ENSMUSG00000046080 | Clec9a | 1.633077 | 1.76E-05 | 0.008559 |
| ENSMUSG00000039699 | Batf2 | 1.539473 | 0.000305 | 0.045964 |
| ENSMUSG00000003484 | Cyp4f18 | 1.521022 | 5.91E-05 | 0.020511 |
| ENSMUSG00000025270 | Alas2 | 1.4461 | 0.000394 | 0.049817 |
| ENSMUSG00000028931 | Kcnab2 | 1.434878 | 1.76E-05 | 0.008559 |
| ENSMUSG00000032420 | Nt5e | 1.383765 | 6.64E-05 | 0.02061 |
| ENSMUSG00000035373 | Ccl7 | 1.3751 | 0.000277 | 0.044945 |
| ENSMUSG00000028268 | Gbp3 | 1.334493 | 1.43E-13 | 2.43E-09 |
| ENSMUSG00000098188 | Sowahc | 1.319119 | 0.000137 | 0.032433 |
| ENSMUSG00000040345 | Arhgap9 | 1.31433 | 2.44E-05 | 0.011523 |
| ENSMUSG00000079547 | H2-DMb1 | 1.305974 | 5.00E-08 | 0.000106 |
| ENSMUSG00000031712 | Il15 | 1.260569 | 3.33E-06 | 0.002575 |
| ENSMUSG00000016496 | Cd274 | 1.209345 | 3.99E-05 | 0.01621 |
| ENSMUSG00000030223 | Ptpro | 1.200504 | 6.02E-05 | 0.020511 |
| ENSMUSG00000004266 | Ptpn6 | 1.199974 | 8.58E-08 | 0.000162 |
| ENSMUSG00000067212 | H2-T23 | 1.181445 | 4.75E-07 | 0.000623 |
| ENSMUSG00000000682 | Cd52 | 1.161262 | 0.000352 | 0.04796 |
| ENSMUSG00000055413 | H2-Q5 | 1.158198 | 0.000198 | 0.037922 |
| ENSMUSG00000024338 | Psmb8 | 1.147643 | 2.83E-05 | 0.012689 |
| ENSMUSG00000037649 | H2-DMa | 1.146587 | 1.07E-05 | 0.006256 |
| ENSMUSG00000039899 | Fgl2 | 1.146013 | 1.90E-08 | 5.50E-05 |
| ENSMUSG00000028270 | Gbp2 | 1.127886 | 1.56E-05 | 0.008036 |
| ENSMUSG00000049103 | Ccr2 | 1.125235 | 7.26E-05 | 0.02061 |
| ENSMUSG00000079363 | Gbp4 | 1.120337 | 6.32E-05 | 0.020555 |
| ENSMUSG00000030657 | Xylt1 | 1.114863 | 4.49E-06 | 0.003325 |
| ENSMUSG00000031897 | Psmb10 | 1.072655 | 2.92E-07 | 0.000451 |
| ENSMUSG00000073409 | H2-Q6 | 1.072607 | 9.50E-05 | 0.024511 |
| ENSMUSG00000064262 | Gimap8 | 1.066711 | 7.05E-06 | 0.004802 |
| ENSMUSG00000073421 | H2-Ab1 | 1.060915 | 5.27E-09 | 2.25E-05 |
| ENSMUSG00000037321 | Tap1 | 1.053361 | 1.33E-06 | 0.001192 |
| ENSMUSG00000026395 | Ptprc | 1.04964 | 1.12E-05 | 0.006336 |
| ENSMUSG00000079227 | Ccr5 | 1.046714 | 0.000173 | 0.035412 |
| ENSMUSG00000036594 | H2-Aa | 1.035383 | 9.13E-07 | 0.000864 |
| ENSMUSG00000035929 | H2-Q4 | 1.030949 | 3.37E-07 | 0.000478 |
| ENSMUSG00000024610 | Cd74 | 1.029912 | 8.94E-07 | 0.000864 |
